# Chiropractic care of Parkinson's disease and deformity

**DOI:** 10.25122/jml-2021-0418

**Published:** 2022-05

**Authors:** Eric Chun-Pu Chu, Alan Te-Chang Chen, Ricky Chiang

**Affiliations:** 1. New York Chiropractic and Physiotherapy Centre, EC Healthcare, Hong Kong SAR, China; 2. School of Health and Rehabilitation Sciences, University of Queensland, St. Lucia, Australia

**Keywords:** Parkinson's, chiropractic therapy, striatal deformity, PD – Parkinson's Disease, PET – Positron emission tomography, PIP – Proximal interphalangeal, MCP – metacarpophalangeal, COG – Center of gravity, TrMS – Transcranial magnetic stimulation, PDQ – PD Questionnaire, MRI – Magnetic resonance image, SH – striatal hand.

## Abstract

Parkinson's disease (PD) is a progressive neurological disease characterized by muscle stiffness, tremor, slowness of movement, and difficulties with posture and walking. Muscle and joint pain are frequent non-motor symptoms of PD. Pain associated with PD is mainly caused by a combination of truncal dystonia, stooped posture, and muscle rigidity. However, PD deformities were rarely discussed in the literature. A 68-year-old Asian female with PD treated with Levodopa for six years complained of progressive neck pain, contractures, and subluxation of both hands in the last two years. A positron emission tomography (PET) scan revealed decreased rostrocaudal gradient uptake in both posterior putamen. After 9 months of multimodal chiropractic rehabilitation, the patient had significant improvement in symptoms, including pain resolution as per the numeric rating scale and physical and mental improvement as per the PD questionnaire. Radiographic measurement showed significantly improved postural alignment and stability. Measurement of joint motion and angles showed an improvement in hand deformity. Although PD is a neurodegenerative disease that is not curable, multimodal rehabilitation may improve neurological and musculoskeletal functions by inducing proprioceptive balance, motor strength, and joint movement. The current study may illustrate multimodal rehabilitation addressing orthopedic deformity associated with symptoms in a PD patient.

## INTRODUCTION

Parkinson's disease (PD) is a neurodegenerative disease that progressively affects the old population, and its prevalence continues to increase as senior populations grow [1]. Although PD mainly affects movement, pain is a prevalent non-motor symptom of PD, affecting 40% to 75% of patients and substantially influences the patients' quality of life [2]. Compared to the healthy population, patients with PD have significantly higher pain levels and a higher prevalence of discomfort associated with abnormal pain perception modulated by PD [3]. Moreover, PD patients with motor symptoms, including stooped posture, muscle rigidity, and festinating gait, have more frequent and severe pain [4, 5] as compared to patients only with tremors [4].

Although pharmaceuticals are primarily used to treat the motor symptoms of PD, their clinical value tends to diminish over time, frequently due to adverse effects such as dystonia [1]. Similarly, pharmaceuticals such as dopaminergic medications, analgesics, and antidepressants are routinely used to treat the pain associated with PD [5]. In addition, physical therapy has also been performed to mitigate PD-associated pain [5]. Pain management in PD patients may also be beneficial for increasing neurological and musculoskeletal functions by boosting proprioceptive balance, muscular strength, and joint movement.

Here we present a case of idiopathic PD with neck and hand deformities. Conservative treatments not only alleviated musculoskeletal discomfort and improved neurological function but also resulted in a more stable deformity, improved postural alignment, and stability. The current study demonstrates how multimodal chiropractic therapies can be used to correct deformities linked with pain in a patient with PD. Nonpharmacological therapies are frequently required for a specific indication when they might assist the individual patient. It is critical to consider the potential involvement of a multidisciplinary approach in PD.

## CASE REPORT

A 68-year-old Asian woman presented with idiopathic PD and associated neck pain, bilateral hand resting tremor and cogwheel type rigidity for 6 years. Initially, she was treated with Levodopa/carbidopa, but the cogwheel rigidity persisted. Pramipexole was initiated to alleviate the remaining rigidity, but the symptoms became worse. She gradually could not open and close her palms due to stiffness, rigidity, and pain. In the past 2 years, the pain in her neck and rigidity of her hands gradually worsened, limiting her daily activity and negatively impacting her sleeping quality. Her orthopedic, rheumatology, and neurology examinations revealed left-side dominated tenderness in the neck, wrists, and hands. The brain magnetic resonance image (MRI), brain positron emission tomography (PET) scan, electrophysiological examinations of bilateral upper limbs, and blood tests were normal except for diminished rostrocaudal gradient in both posterior putamen at the brain PET scan. The radiological findings are consistent with the diagnosis of idiopathic PD. She received steroid treatment for 6 months and botulinum toxin injection for 1 time and only achieved minimal improvement. The neck pain and striatal hand (SH) deformities had progressively worsened. She was referred to chiropractic rehabilitation for posture-related musculoskeletal pain.

**Figure 1:**
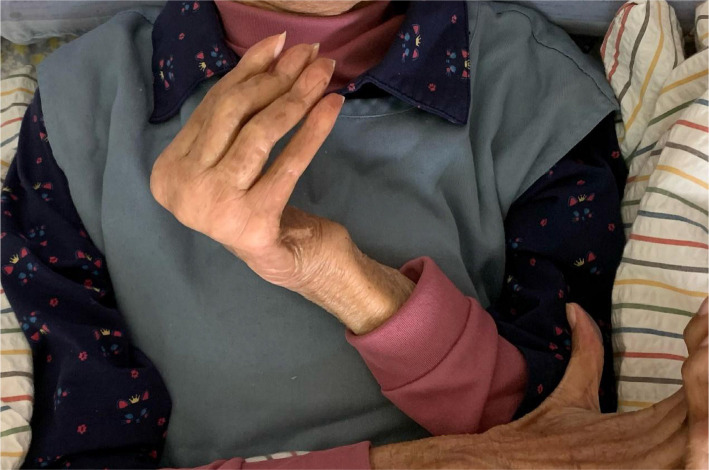
Stage 4 striatal left-hand deformity in a patient with PD for 6 years. Severe bone scintigraphy showed her left metacarpal phalangeal (MCP) at 100° hyperextension, proximal interphalangeal (PIP) at 30° hyperextension, and swan neck deformity of fingers.

**Figure 2:**
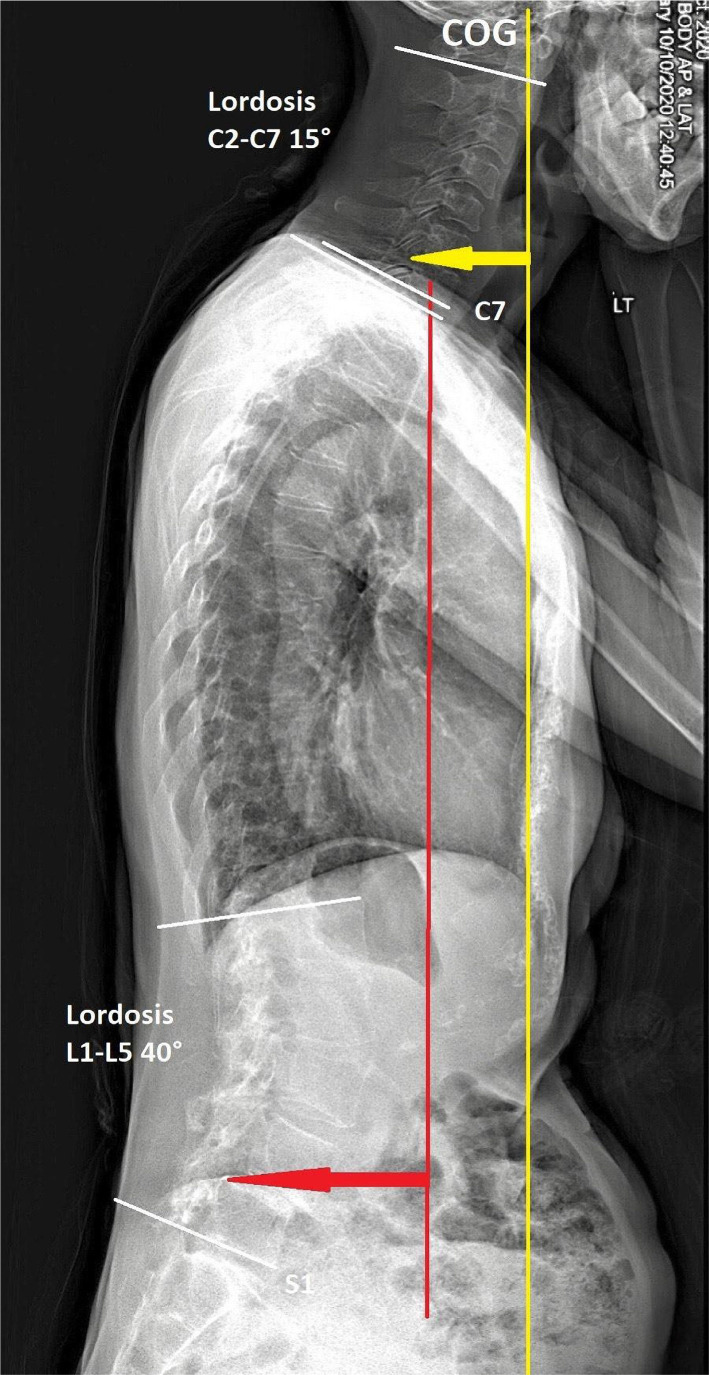
Postural parameters assessed on a sagittal standing full-spine EOS® x-rays: Initial radiograph identified a forward head, stooped posture, and reduced cervical lordosis. The center of gravity (COG) of the head was shifted anteriorly.

**Investigation:** She presented with a stoop and drop-head posture but with no symptoms of autonomic dysfunction, cognitive deterioration, or cerebellar abnormalities on examinations. The muscular palpation revealed stiffness in the bilateral upper trapezius, scalene, and paraspinal muscle in the cervical region. Contractures and subluxations were observed in both hands of the patient who could not open and close the palms, more severely in the left hand (Figure 1). Severe bone scintigraphy showed her left metacarpal phalangeal (MCP) at 100° hyperextension, proximal interphalangeal (PIP) at 30° hyperextension, and swan neck deformity of the fingers. Her right hand showed PIP at 20° hyperextension and swan neck deformity of phalanges. Her motor strength in the hands was diminished, and atrophy was observed in her left hand. A full-spine EOS radiograph (Figure 2) in the weight-bearing position revealed typical PD characteristics, including a forward head, stooped posture, reduced lumbar lordosis, flexed hips and knees joints, and the center of gravity (COG) of the head shifted anteriorly.

**Diagnosis:** With a positive finding of the diminished rostrocaudal gradient in both posterior putamen, the patient was diagnosed with idiopathic PD.

**Treatment:** A series of multimodal chiropractic rehabilitation therapies were performed, including electromagnetic stimulation therapy to hypertonic muscles, spinal manipulation (NYMG^®^ Scoliosis Technique) to alleviate intervertebral and synovial joint restrictions, and high-frequency transcranial magnetic stimulation (TrMS) at 18,000–20,000 pulses over the primate cerebral cortex that contributes to the control of movement to stimulate motor strength. After six consecutive days of daily treatment, the patient's neck pain, upper limb pain, finger movement, and insomnia symptoms have improved significantly. As part of the second phase of treatment, intermittent robotic spinal decompression (WIZ Medical Co., Korea) was applied as vertebral traction. Custom-orthotics were prescribed to stabilize the deformity, and she received therapy three times weekly.

**Figure 3:**
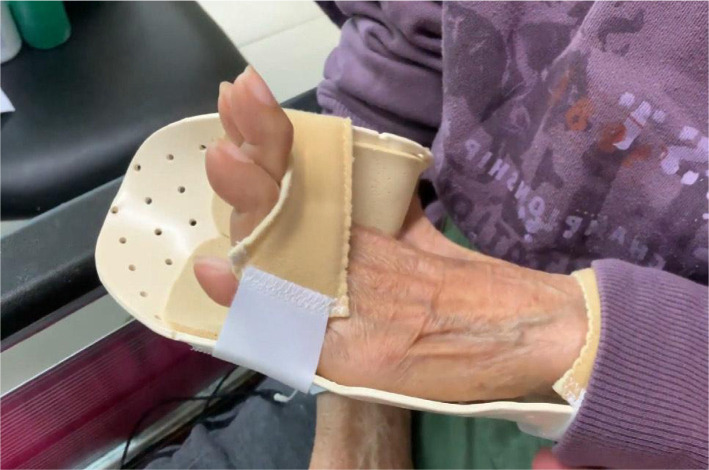
A repeat photograph 9 months later showing substantial improvement in most of the orthopedic parameters. Her left MCP deformity had improved to 30° extension, PIP at 10° extension, and swan neck deformity of fingers remained the same.

**Figure 4:**
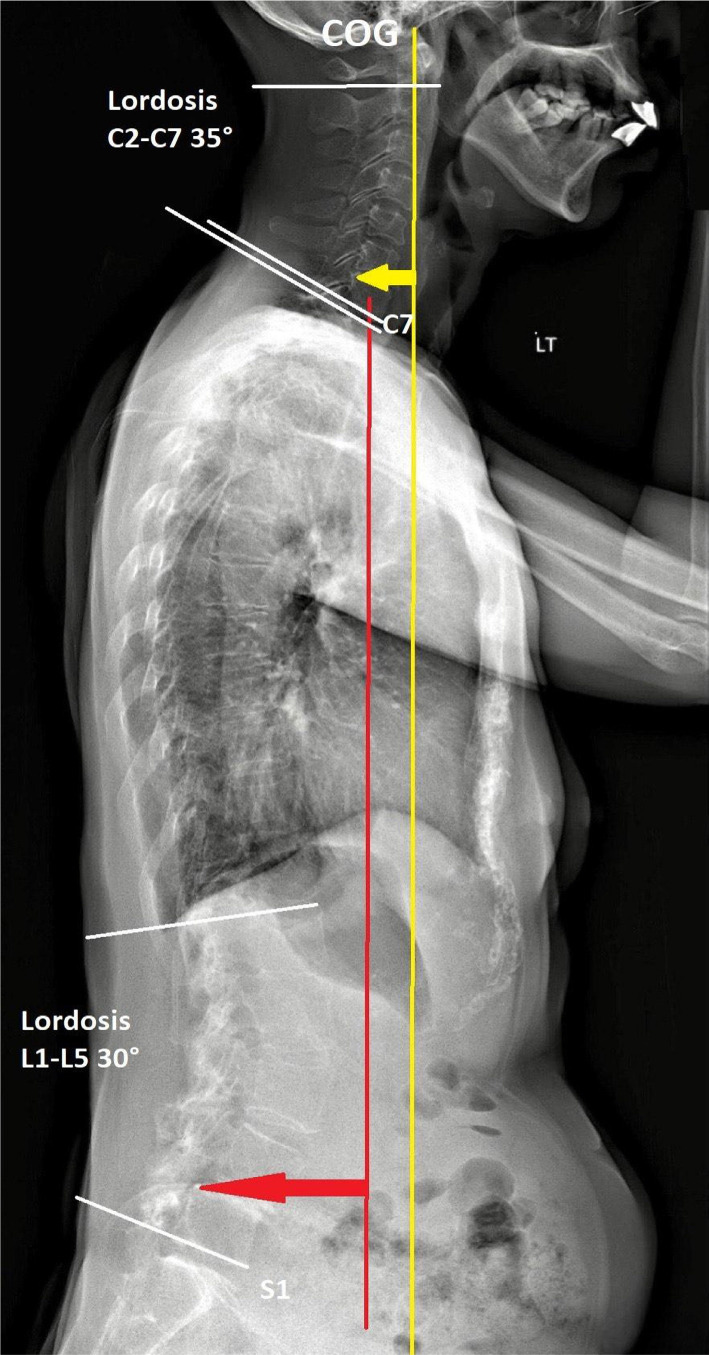
A repeat radiograph taken nine months later demonstrates significant improvement in the majority of the spinal deformity. The C7 plumb line (red line) is drawn caudally from the C7 vertebra's center. The line should be parallel to or within 5 mm of S1's superior-posterior endplate. In a well-aligned subject, the line of COG (yellow line) has improved in the sagittal plane.

**Outcome:** At 9 months after the first consultation, most of her symptoms had significantly improved, including pain relief (numeric pain scale decreased from 8 to 2 on a 0 to 10 scale), PD Questionnaire (PDQ) scores (scores 68 reduced to 32 from 0 to 100 scale), improved left-hand deformity (Figure 3) and postural stability (Figure 4). Although the striatal deformity of the hands was significantly improved, the pain symptoms were reduced without any side effects associated with the treatment. No adverse effect was reported during the treatments.

## DISCUSSION

Pain is a frequent non-motor symptom in PD that significantly changes the patients' living style. Pain associated with PD is frequently felt in the neck, upper back, and lower limbs, which are most likely caused by physical exertion such as drop head syndrome, truncal dystonia, hyperkyphotic thoracic spine, and festinating gait [2]. Pain may also be caused by common orthopedic comorbidities associated with aging, including osteoporosis, osteoarthritis, and muscle atrophy [6, 7]. However, around eight to forty percent of patients with PD reported experiences of discomfort years before they identified any of the parkinsonian symptoms [7, 8]. Thus, in the early stages of PD, sensory pain is not a comorbidity of parkinsonian motor impairment [6]. PD alters the neurological and chemical systems involved inside the central nervous system in processing and modulating pain perception [4]. Early structural change in the lower brainstem and the nuclei at the peripheral nervous system, particularly the autonomic and enteric ganglia, is marked as the pre-motor period [7]. Small-fiber neuropathy associated with idiopathic PD may lead to aberrant pain perception, according to pathological observations of neuritic deposition of presynaptic neuronal protein (α-synuclein) in skin denervation and peripheral nerve fibers [9]. In conclusion, it is believed that the degeneration process of the peripheral nerve may lead to nociceptive dysfunction of the central nervous system at the early PD [6].

The effects of motor and nociceptive limitations can manifest themselves in a variety of ways, including bodily dysfunction, melancholy, illness-related lifestyle, and social behaviors. A central nervous system agent, Levodopa, works by being converted to dopamine in the brain in treating neurological symptoms of PD. Levodopa has been shown to neutralize the pain-activating agents of nociceptive pathways, consequently leading to increased pain thresholds in PD [5]. Tricyclic antidepressants and anticonvulsants are used as an alternative for PD-related peripheral neuropathy in patients who do not respond well to Levodopa [6]. However, the existing data support that the use of non-steroid anti-inflammatory medicines or analgesics are inadequate for pain relief in PD patients [10].

Musculoskeletal pain, central pain, neuropathic pain, akathisia pain, and dystonia-related pain are the 5 types of pain in patients with PD [10, 11]. Among the numerous pathways underlying PD-related pain, musculoskeletal pain accounts for 40 to 90 percent of reported symptoms [10]. For musculoskeletal pain, complementary therapies are advocated, including physiotherapeutic and chiropractic treatments, focused on pain management, motor strengthening, joint mobility, and postural training exercise [10, 12].

Rigidity, like a tightness in the limbs, is highly correlated with pain in PD [4]. Rigidity also contributes to musculoskeletal discomfort by disrupting normal posture, resulting in stiffness, decreased flexibility, and changed body mechanics [4]. The striatal hand is one of the most understudied postural anomalies in PD [13]. It leads to extreme abnormalities of hand posture, causing altered dexterity, pain, and disfigurement [13]. Flexion at the metacarpophalangeal joints, extension at the interphalangeal joints, and ulnar deviation are common characteristics of the SH [14]. The misdiagnosis of the deformities of PD frequently occurs in the early stage of PD in the absence of tremor, bradykinesia, and rigidity [15]. They are often diagnosed as orthopedic symptoms, given their similarity to rheumatoid arthritis [15]. However, it is distinguishable from inflammatory arthritis deformity, given that PD deformity has no swelling. They are also often misdiagnosed as levodopa-induced and primary dystonia, which is distinguishable because the PD deformity persists throughout sleep [14]. Although the exact mechanism is unknown, it is hypothesized that stiffness contributes to limb abnormalities [14].

As the facilitatory and inhibitory pathways in the nervous system affecting parkinsonian rigidity and hypokinesia are not well understood [16], there is no agreed standard for determining how far PD has progressed. To manage motor impairments in patients with PD, non-invasive treatments including physical rehabilitation, osteopathic manipulation, deep brain stimulation, and transcranial magnetic stimulations (TrMS) are frequently applied. The high-frequency repetitive TrMS, particularly when delivered bilaterally over cortical motor regions, has been shown to improve parkinsonian motor symptoms [17]. In addition, physical rehabilitation also showed improvement in motor function in patients with PD in clinical investigations [18]. A chiropractic manipulation program [19] or even a single osteopathic therapy session [20] showed promising outcomes in recovering motor functions. Similarly, various therapies have been used to treat musculoskeletal conditions, emphasizing mobilizing restricted structures, relieving neurological compromise, and optimizing the function of constricted joints and afflicted muscles [21, 22].

Our case showing reduced discomfort and deformity in SH using physical interventions is consistent with prior reports demonstrating the potential of chiropractic treatments for patients with PD [23, 24]. We speculate that the custom-designed orthotic of the left hand may have improved the treatment prognosis by stabilizing the joints deformity, the TrMS improved PD-freezing of the hands by normalizing brain connectivity [25], and chiropractic manual therapy may have enhanced muscular proprioception, motor functioning, and postural balance [22]. Accordingly, multimodal treatments for patients with PD resulted in significant improvements for both posture and hand deformities. The limitations of the study include a lack of a control group or a small sample size. However, the study provides evidence on how chiropractic can prove parkinsonian symptoms.

## CONCLUSION

Our study illustrates reduced discomfort and deformity in SH using chiropractic rehabilitation for patients with PD. The striatal hand (SH) is one of the most understudied postural anomalies in Parkinson's disease (PD). It causes severe hand postural anomalies, resulting in decreased dexterity, discomfort, and deformity. Multimodal intervention addressing posture and deformity issues associated with pain is crucial in the management of PD.
